# GaitRGA: Gait Recognition Based on Relation-Aware Global Attention

**DOI:** 10.3390/s25082337

**Published:** 2025-04-08

**Authors:** Jinhang Liu, Yunfan Ke, Ting Zhou, Yan Qiu, Chunzhi Wang

**Affiliations:** 1 School of Computer Science, Hubei University of Technology, Wuhan 430068, China; liujinhang@hbut.edu.cn (J.L.); keyunfan@hbut.edu.cn (Y.K.); 102411198@hbut.edu.cn (T.Z.); chunzhiwang@hbut.edu.cn (C.W.); 2 Department of Computer Science, College of Engineering and Technology, Hubei University of Technology, Wuhan 430068, China

**Keywords:** GaitRGA, biometric identification, silhouette-based Gait recognition, deep learning, neural network

## Abstract

Gait recognition, a long-range biometric technique based on walking posture, the fact that they do not require the cooperation of the subject and are non-invasive has made them highly sought after in recent years.Although existing methods have achieved impressive results in laboratory environments, the recognition performance is still deficient in real-world applications, especially when confronted with complex and dynamic scenarios. The major challenges in gait recognition include changes in viewing angle, occlusion, clothing changes, and significant differences in gait characteristics under different walking conditions. To slove these issues, we propose a gait recognition method based on relational-aware global attention. Specifically, we introduce a Relational-aware Global Attention (RGA) module, which captures global structural information within gait sequences to enable more precise attention learning. Unlike traditional gait recognition methods that rely solely on local convolutions, we stack pairwise associations between each feature position in the gait silhouette and all other feature positions, along with the features themselves, using a shallow convolutional model to learn attention. This approach is particularly effective in gait recognition due to the physical constraints on human walking postures, allowing the structural information embedded in the global relationships to aid in inferring the semantics and focus areas of various body parts, thereby improving the differentiation of gait features across individuals. Our experimental results on multiple datasets (Grew, Gait3D, SUSTech1k) demonstrate that GaitRGA achieves significant performance improvements, especially in real-world scenarios.

## 1. Introduction

**Figure 1:**
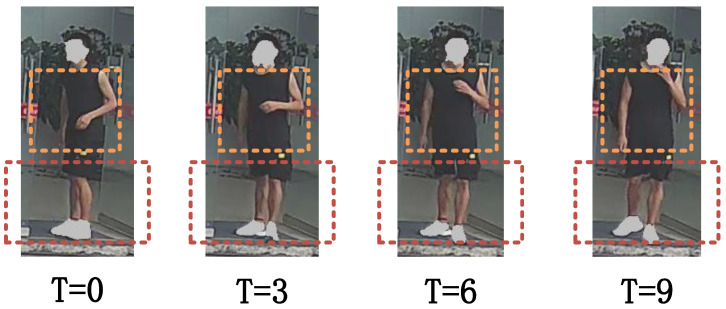
Subjects’ greatest changes per exercise were in the hands and legs.

Gait recognition [1,2], a biometric identification method that leverages unique walking patterns, has attracted considerable attention in recent years within the field of biometrics. Compared with other biometric modalities [3,4], gait recognition stands out for its ability to be captured effortlessly from a distance without physical contact, making it particularly advantageous for applications in surveillance and security prevention, which makes it particularly suitable for applications in the fields of surveillance and security prevention. Gait recognition is characterized by non-intrusiveness and is particularly suitable for complex non-cooperative environments. Therefore, it has in depth research in scenarios such as public safety, identity verification, and criminal investigation [5]. With the advancement of deep learning, researchers have started leveraging deep learning methods to address these challenges [6,7,8,9,10,11,12,13,14]. However, there are still many challenges in practical application scenarios, especially in complex real-world scenarios where the model is often affected by multiple external factors. For example, factors such as viewpoint changes, occlusion, clothing changes, and carrying objects can negatively affect the model. These factors lead to increased diversity and uncertainty in gait features, and the robustness of gait recognition is difficult to ensure. As shown in Figure 1, the dynamic changes of gait in complex scenes and the independent motion features in localized regions of the human body bring great challenges to feature extraction.

**Figure 2:**
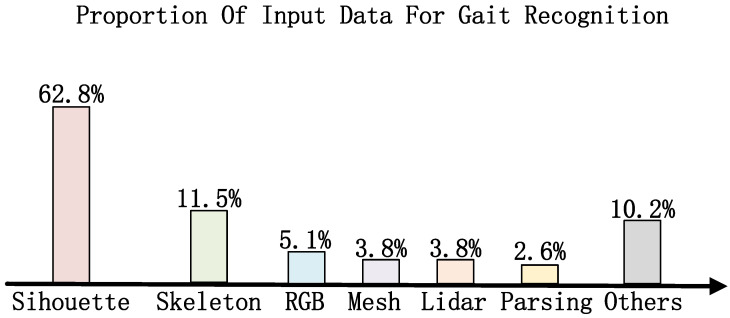
Types of different input data.

Existing gait recognition methods are generally divided into three primary categories: silhouette-based methods, skeleton-based methods, and multi-modal fusion-based methods. As shown in Figure 2, the silhouette-based approach is one of the most commonly used methods, relying on extracting the silhouette features of the human body shape for modeling, and is especially good at modeling the overall dynamic features of gait. This approach has some challenges when dealing with complex scenes, such as viewpoint changes and occlusion, but its advantages in overall dynamic modeling are still obvious. Skeletal-based methods, on the other hand, perform feature extraction through human key points or skeleton maps, and although they perform better with clothing changes or partial occlusion, they have limited ability to capture detailed features and rely on high-quality key point detection. Multi-modal-based approaches combine multiple data sources (e.g., silhouette and skeleton) to further enhance model robustness and accuracy by integrating information from different modalities to capture gait features more comprehensively. However, multi-modal approaches impose higher requirements on data acquisition and processing, and the synchronization and fusion of data also increase system complexity and computational cost.

Recent research has concentrated on enhancing gait feature representations and improving model robustness in handling complex and dynamic scenarios.For example, GaitGL [15] achieved effective extraction of overall and local features by combining global convolution and local feature masking mechanisms, thus significantly improving the differentiation ability of the model. GaitPart [16] divides the human body into different parts and independently extracts the spatial and temporal representations for each part, enabling it to model short-term temporal dependencies. However, it overlooks the interconnectedness and joint movement of body parts during walking. In addition, GaitMGL [17] integrates global-local spatial features and multi-scale temporal features to effectively capture motion patterns, improving gait recognition accuracy across diverse condition. These studies suggest that the improvement of gait recognition performance lies in the effective extraction and fusion of multiple features to cope with complex changes in real scenes.

To tackle the aforementioned challenges, this paper proposes the GaitRGA. Our GaitRGA module provides clustering-like information about the correlations between spatial locations in gait silhouettes, which is extremely helpful for inferring semantics and attention, particularly in gait images where human poses may be constrained. By explicitly exploring global relationships to mine structural information, we enable the attention mechanism to focus on discriminative body regions within the gait silhouette. Specifically, for each feature node (e.g., a feature vector at a spatial position on the feature map), we model the pairwise relations of this node with all other nodes and compactly stack these relations into a vector (representing global structural information). This vector is then combined with the node’s own feature to infer the attention strength via a compact model. This approach takes into account both the appearance features and their global relationships, determining feature importance from a global perspective.

Our main contributions can be summarized as follows:

1. We propose a method to determine feature importance from a global perspective by leveraging the appearance features and their global relationships. For each feature node, we build a compact representation by stacking its pairwise relations with all other nodes into a vector, and mine patterns from this representation to learn attention.

2. We introduce a relation-aware global attention mechanism, which compactly represents the global scope relations and infers attention based on these relations through two convolutional layers. We apply this design to both spatial (RGA-S) and channel (RGA-C) dimensions and demonstrate its effectiveness in gait recognition tasks.

## 2. Related Work

### 2.1. Silhouette Based Gait Recognition

Silhouette-based methods are widely used in gait recognition due to their effectiveness in extracting morphological features of human silhouettes and capturing comprehensive movement patterns, making them highly practical across diverse application scenarios [18,19]. Among them, GaitSet [20] is a pioneering study, which for the first time modeled gait sequences as sets and improved the recognition accuracy by extracting global features. Its advantage lies in its efficient modeling of overall features, but the descrip tion of local feature changes is not fine enough, leading to its limited performance in complex environments. To improve this deficiency, GaitPart [16] proposes a method based on GaitSet [20] to partition human gait into multiple parts for independent modeling, which enhances the ability to capture fine-grained features, and especially performs more robustly in the case of view angle changes and occlusion. GaitPart [16] significantly improves the capture of the the capture of motion details of human body parts, especially the performance in complex scenes is significantly improved. Subsequently, GaitEnergy Image (GEI) [21] successfully captured the overall dynamic information of gait by fusing multi-frame gait images into a gait energy map, which improved the stability and consistency of the model on gait features.Although GEI [20] has a great advantage in capturing the overall dynamic information, the modeling of features in the temporal dimension is still insufficient. To address this problem, Chrono-Gait Image (CGI) [22] introduces the temporal dimension and divides the gait sequence into different time segments, which better models the temporal dynamic characteristics of gait and enhances the description of gait behavior details. These methods have gradually made progress in modeling silhouette features, providing richer feature representations for subsequent gait recognition studies.

### 2.2. Skeleton Based Gait Recognition

Skeleton-based methods represent the human body using key points or skeleton maps, emphasizing the movement trajectories of different body parts, making them particularly effective for gait recognition in scenarios involving clothing variations or occlusions [21]. PoseGait [23] is one of the important advances in skeleton-based methods, which significantly reduces the effect of clothing changes on gait recognition by modeling human posture using 3D keypoint information combined with a prioriknowledge, leading to more consistent performance across different clothing conditions. To further enhance the representation capability of skeleton features. GaitGraph [24] introduces graph convolution network (GCN), which captures the relationship between different joints by modeling a 2D skeleton graph, thus improving the expressive ability and recognition accuracy of skeleton features, and is especially good at capturing dynamic behaviors.SkeletonGait [25] focuses on the dynamic modeling of the human body’s dynamic modeling of key points, and captures the motion trajectories of joints by introducing temporal information, which significantly improves the robustness in complex scenes. DyGait [26] further utilizes the skeleton information for modeling, which provides the ability to capture the three-dimensional features of gait, and thus provides a richer expression of features for gait recognition, which leads to a better expressiveness in dealing with complex environments.

### 2.3. Multi-Modal Based Gait Recognition

Recently, multi-modal approaches have emerged as a prominent research focus in the area of gait recognition, which combine multiple data sources, such as silhouette, skeleton, and depth information, in order to comprehensively capture gait features and enhance the robustness and accuracy of the model.HybridGait [18] is one of the representative works in the multi-modal approach, which combines multi-modal features by combining 3D Silhouette and 2D silhouette information for effective integration, which addresses the challenges of multiple viewpoints and clothing changes, thus enhancing the model’s adaptability in complex environments. Combining gait silhouette features with time series features enhances the model’s ability to capture both static information and dynamically changing features of gait. especially in diverse environments. Subsequently, MMGait [27] further improved the model’s recognition performance across viewpoints and complex backgrounds by fusing multi-modal data such as RGB, depth and infrared images. In particular, in the process of fusing multiple spectral information, the model’s performance in dealing with scenes with complex backgrounds and illumination variations is made even better by the careful modeling of each modal feature. These multi-modal approaches have demonstrated notable success in feature fusion, leading to a substantial improvement in the model’s ability to handle complex scenes effectively.

## 3. GaitRGA

**Figure 3:**
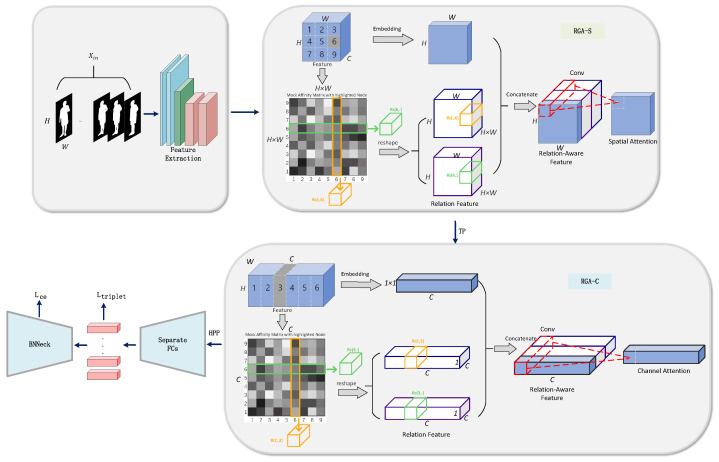
GaitRGA’s Overall Process.

Our designed gait recognition network, illustrated in Figure 3, first extracts features from the gait silhouette frames using a backbone network (ResNet-like), which converts the input multi-frame gait Silhouette sequences into a 3D feature map. RGA-S extracts features through edge enhancement to obtain edge weights, and multiplies the input features with the edge weights. Meanwhile, it computes spatial relationships and reshapes the spatial relationship matrix. Then, local features are extracted, and these local features are concatenated with spatial relationship information. Finally, the spatial network generates weights, which are ultimately applied to the features, the Time Pooling (TP) module applies max pooling along the temporal dimension to extract the feature mapping sequence and obtain global information. Next, RGA-C extracts channel weights through channel enhancement and multiplies the input features with the channel weights. Meanwhile, it computes channel relationships and reshapes the channel relationship matrix. Then, local features are extracted, and these local features are concatenated with channel relationship information. Finally, the channel network generates weights, which are ultimately applied to the features. The aggregated features are partitioned into multiple local region feature vectors by the Horizontal Pyramid Pooling (HPP) module, with each vector independently mapped to the metric space via a fully connected layer, resulting in high-dimensional embeddings. Finally, the feature distribution is refined using BNNecks, and the model is jointly optimized through ternary loss and cross-entropy loss.

(1) $$F_{E}=f\left(x_{i}\right)$$

(2) $$X_{out}=H_{BN}\left(G_{FC}\left(F_{p}\left(F_{\alpha }\left(F_{E}\right)\right)\right)\right)$$

The f(·) in equation denotes the ResNet-Like feature extractor, which serves to extract the feature $x_{i}$ from the silhouette gait sequence. $F_{\alpha }\left(\cdot \right)$ denotes the RGA-S, $F_{\beta }\left(\cdot \right)$ denotes the RGA-C, $G_{\mathrm{FC}}\left(\cdot \right)$ stands for the Fc, and $H_{\mathrm{BN}}\left(\cdot \right)$ stands for the BNNecks.

### 3.1. Resnet-like

**Figure 4:**
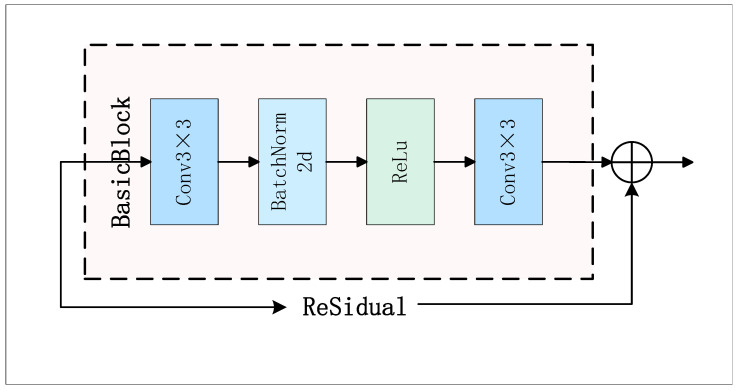
Residual structure for each layer.

The development of CNN architectures has progressed from shallow to deep networks, leading to the introduction of outstanding backbone models like AlexNet, VGG-16, and ResNet [28,29,30]. However, previous work in gait recognition [18,31,32,33,34,35,36] relied heavily on ordinary convolutional neural networks consisting of a few basic convolutional layers. In GaitRGA, we refer to the class ResNet proposed by GaitBase [33] as our feature extractor. The structure of this network is illustrated in Table 1, where a 64 × 44 sized input feature map is input, and the number of channels is first extended to 32 channels by a 3 × 3 convolutional layer, followed by the BatchNorm2d and ReLU activation functions, the feature dimension is kept as (N, 32, 64, 44). The backbone part of the network consists of four layers, as shown in Figure 4, and each layer contains a different number of residuals; Layer1 keeps the feature dimension unchanged; Layer2 and Layer3 are downsampled by convolution with stride = 2, and at the same time the number of channels is doubled, so that the feature map dimensions gradually changed to 32 × 22 and 16 × 11; the final Layer4 increases the number of channels to 256 and the output dimension is (N, 256, 16, 11). The whole process can be expressed mathematically as:

(3) $$x_{0}=\mathrm{ReLU}\left(\mathrm{BN}\left(W_{0}\cdot x+b_{0}\right)\right)$$

Assuming that the input is characterized by *x*, $x_{0}$ is obtained through activation operations after convolution and batch normalization. Where $W_{0}$ and $b_{0}$ are the convolution kernel and the bias term respectively, and · represents the convolution operation. Batch normalization is used to standardize the output after convolution to speed up training and improve model stability.

(4) $$y=\mathrm{ReLU}\left\{F\left(x,\{w_{i}\}\right)+x\right\}$$

The formula describes the process of calculating residual blocks. $F(x,\left\{w_{i}\right\})$ represents the result of the convolution operation on input *x*. The convolution kernel set is $\left\{w_{i}\right\}$. The input *x* and the convolution result $F(x,\left\{w_{i}\right\})$ are added together and processed by the ReLU activation function.

(5) $$y=\mathrm{Layer4}\left(\mathrm{Layer3}\left(\mathrm{Layer2}\left(\mathrm{Layer1}\left(x_{0}\right)\right)\right)\right)$$

### 3.2. Relation-Aware Global Attention

**Figure 5:**
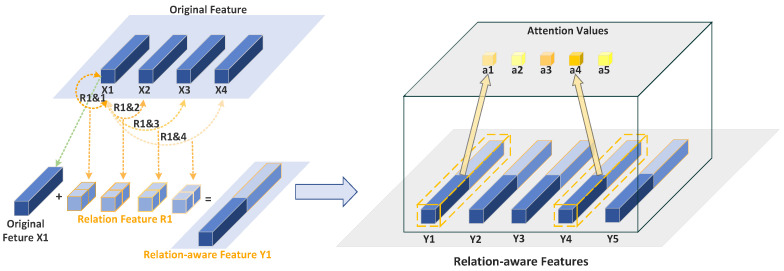
Proposed relation-aware global attention: Learn attention by taking into account the global relation information. For the *i*-th (here i=1) feature vector, the global scope relation information is represented by stacking the pairwise relations $r_{i}=\left[r_{i,1},\cdots ,r_{i,5},r_{1,i},\cdots ,r_{5,i}\right]$.

RGA enhances the model’s focus on important features by capturing the relationships between features across the entire global scope. Specifically, as shown in Figure 5, it considers the relationships between each feature point and all other feature points, combining this relational information with the local features at that point, thus learning a more accurate attention weight for each feature point. The core idea of this method is to treat the pairwise relationships between features as global structural information, stacking these relations to create a comprehensive feature representation, which is then used by a shallow convolutional network to infer the importance of each feature point. In this way, RGA effectively understands the structural information in the image from a global perspective, allowing it to better select features that are useful for gait recognition tasks, while suppressing irrelevant distractions.

### 3.3. RGA-S

**Figure 6:**
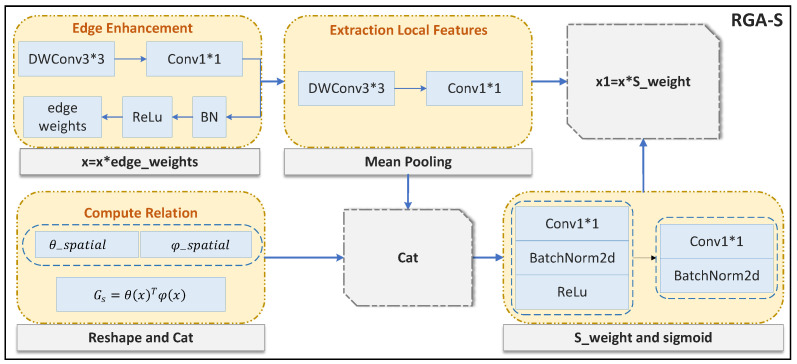
Specific implementation details of RGA-S.

RGA-S optimizes feature representation in gait recognition by means of global spatial relationships. The core idea is to capture spatial relationships between features to improve the focus on key features. As show in Figure 6. Assuming the input feature is *X*, it first undergoes edge enhancement, where depthwise separable convolutions (DWConv3 × 3) and point convolutions (Conv1 × 1) are applied to output the edge enhancement weights. These weights are then multiplied with *X*, resulting in $X_{1}$:

(6) $${\tilde{X}}_{1}=\left[{\mathrm{pool}}_{c}\left(\psi_{s}\left(x_{i}\right)\right),\phi_{s}\left(r_{i}\right)\right]$$

Among them, $\psi_{s}\left(x_{i}\right)$ represents the input characteristics of the spatial information, which will be introduced to the pooling operation (pooling) for further processing. $\varphi_{s}\left(r_{i}\right)$ is used to calculate the spatial relations between feature points.

At the same time, the spatial relationships are calculated, and the spatial relationship matrix is reconstructed by transposing and concatenating the relations. Then, $X_{1}$ passes through a 3 × 3 depthwise separable convolution and a 1 × 1 convolution, followed by mean pooling to reduce the feature dimensionality to a single channel, minimizing channel interference and improving the representation of local spatial features. The local features and spatial relationship information are then concatenated, and spatial networks generate weights, which are finally applied to the features:

(7) $$a_{i}=\mathrm{Sigmoid}\left(W_{2}\mathrm{ReLU}\left(W_{1}{\tilde{X}}_{1}\right)\right)$$

where $a_{i}$ represents the spatial attention coefficient of the *i*-th feature point, which determines the degree of attention of each feature. σ is the Sigmoid activation function. $W_{1}$ and $W_{2}$ are the weight matrices of the convolution layer for feature conversion and weighting operations.

In this way, RGA-S is able to better capture the global structure in the image and help the model focus on the most discriminative part of the gait image, thus improving the effectiveness and robustness of the gait recognition task.

### 3.4. RGA-C

**Figure 7:**
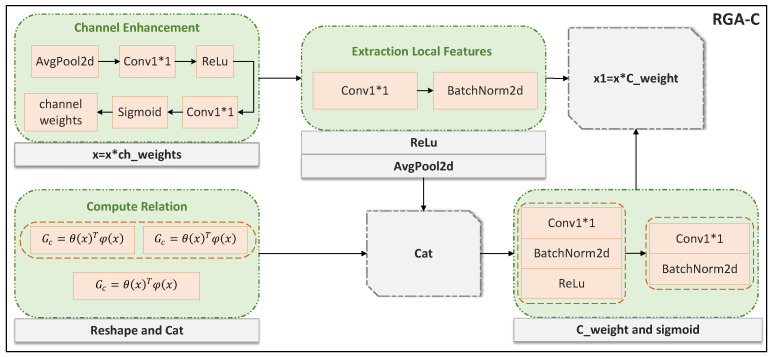
Specific implementation details of RGA-C.

RGA-C is a channel attention mechanism designed for gait silhouette images, focusing on enhancing the channel dimension of feature representations. Assume a feature $X_{i}$, where i=1,2,…,C. As shown in Figure 7, The process begins with a channel enhancement module, which generates channel weights through adaptive average pooling and a series of convolution operations, emphasizing important channel information. The core component is channel relationship modeling, which calculates the similarity matrix between channels to capture the dependencies between them, Similar to spatial relationships, the pairwise relationship $r_{i,j}$ from node *i* to node *j* is defined as an affine transformation in the embedding space:

(8) $$r_{i,j}=f_{c}\left(x_{i},x_{j}\right)=\theta_{c}{\left(x_{i}\right)}^{T}\phi_{c}\left(x_{j}\right),$$

$\theta_{c}\left(x_{i}\right)$ represents the channel information of the feature extraction, and $\varphi_{c}\left(x_{j}\right)$ represents the transform operation of the feature, used to calculate the relationship between $x_{i}$ and $x_{j}$.

To avoid gradient vanishing issues, RGA-C introduces a temperature scaling mechanism when computing the channel attention, dividing by the square root of the feature dimension. Then, RGA-C combines the global features with the channel self-attention and passes them through multiple convolution layers to generate the final channel attention weights. These weights are normalized through a sigmoid function and applied to the original features, enhancing the key channels.

## 4. Exeperiments

This section assesses GaitRGA’s effectiveness using three widely recognized datasets, including Grew [34], Gait3D [32] and SUSTech1k [35]. First, we introduce these datasets and the corresponding training configurations. Subsequently, we conduct comparisons against the most advanced gait recognition methods under identical experimental setups. Finally, we will determine how the various components of GaitRGA affect performance through ablation experiments This section may be divided by subheadings. It should provide a concise and precise description of the experimental results, their interpretation as well as the experimental conclusions that can be drawn.

### 4.1. Datasets

Three popular datasets are used in this paper, covering a diverse range of needs from large-scale field scenarios to laboratory environments. These include the widely used large-scale field datasets Grew [34] and Gait3D [32], and the small-scale gait dataset SUSTech1k [35] released by the Southern University of Science and Technology. Table 2 demonstrates the information on identity and number of sequences contained in each dataset.

Grew [34] serves as a large-scale dataset tailored for gait recognition in real-world scenarios. Designed to address challenges in gait recognition under unconstrained environments, it incorporates diverse and complex variations. Grew [34] encompasses a total of 26,345 identities and 128,671 sequences, which were recorded using 882 cam eras strategically placed in public areas, which providing multiple data types such as silhouettes, poses, and optical flow. The dataset is split into training and test sets, with the training set comprising the majority of sequences and the test set structured for rigorous evaluation. During testing, specific sequences are designated as probes, while the remaining sequences constitute the gallery. With its wide range of occlusions, lighting conditions, multi-view variations, and dynamic environments, Grew [34] serves as an excellent benchmark for evaluating the generalizability of gait recognition models in real-world scenarios.

Gait3D [32] serves as a comprehensive dataset designed specifically for 3D gait recognition. designed to provide a resource for research on dense 3D representations and gait recognition in realistic scenarios. Gait3D [32] is a 39-camera shot at a hypermarket that includes 4000 identity counts and 25,309 sequence counts, and has three data types: 2D, 3D representations, and raw RGB frames. The dataset is divided into training and test sets, where the training set includes 18,940 gait sequences from 3000 identities, while the test set comprises 6369 gait sequences from 1000 identities. During testing, one sequence is chosen at random to act as the probe for each identity, while the remaining sequences constitute the gallery. With its complex occlusions, dynamic backgrounds, viewpoint variations, and gait irregularities, the dataset is ideal for evaluating the adaptability of gait recognition models in real-world scenarios.

SUSTech1K [35] represents a pioneering large-scale dataset for LiDAR-based gait recognition, designed to explore the potential of point cloud data in this field. especially for the application of 3D representations in realistic scenarios. SUSTech1K [35] consists of 1050 identity counts and 25,239 sequence counts, and has three data types: LIDAR (high-precision 3D point cloud data), RGB images (with corresponding silhouette maps), and synchronized frames as three data types. It is separated into a training set, a validation set, and a test set. The training set includes 250 identities with a total of 6011 sequences; the validation set also contains 250 identities with a total of 6025 sequences. and the test set contains 550 identities with 13,203 sequences, and the test data is divided into subsets based on different attributes (e.g., carrying items, clothing changes, occlusion, etc.) during the testing phase.

The official protocol is followed in all our experiments, following the protocol for training, testing, library and probe set partitioning strategies to obtain relevant experimental data, using the main evaluation metric Rank-k (k = 1, 5, 10, 15, 20) [37] to evaluate our experiments. Where Rank-k refers to the probability of correctly identifying the target among the first k candidates in the search result. mAP comprehensively considers the average accuracy under different recall rates. mINP measures a model’s ability to identify the most difficult samples.

### 4.2. Implementation Details

We will present the hyper-parameters used by GaitRGA for the experiments with different datasets, as shown in Table 3, where we used the following settings.

In our experiments, the batch size for training is represented as (N, S), with N indicating the ID (i.e., tag number) and S representing the sequence number per tag. For the Grew and Gait datasets, the batch size is set to (32, 4), while for the SUSTech1K dataset, it is configured as (8, 8).

The SGD optimizer was employed for back propagation, utilizing an initial learning rate of 0.05, a momentum value of 0.9, and a weight decay rate of 0.0005 to ensure effective training and regularization of the model parameters. For the Grew dataset, a factor of 10 reduction in the learning rate is applied at 80 k, 120 k, and 150 k rounds. For the Gait3D dataset, a total of 60 k steps were trained, and the learning rate was reduced by a factor of 10 on rounds 20 k, 40 k, and 50 k. For the SUSTech1K dataset, the learning rate decayed by a factor of 10 on 20 k, 40 k rounds and a total of 50 k rounds were trained.

To reduce the impact of noise, we applied data augmentation techniques on the Grew [34], SUSTech1k [35] dataset, including random perspective transform, horizontal flip, rotational transform, and random erasure with a probability of 20%. This study was conducted on 8 NVIDIA GeForce RTX 1070TI GPUs (64 G video memory) using the PyTorch deep learning framework (1.13.1) with CUDA11.7 in an experimental environment.

### 4.3. Comparison with Other Methods

#### 4.3.1. Evaluate on Grew Dataset

We compare our method with the current SOTA approaches on the large-scale Grew [34]. As shown in Table 4, from experimental results, GaitRGA surpasses all existing approaches, achieving an accuracy improvement of 8.78% over QAGait [19] which is one of the most recent silhouette-based gait recognition methods. The results in the table demonstrate that GaitRGA achieves the highest Rank-1 accuracy of 67.88%, significantly surpassing other methods like GaitSet [20], GaitPart [16] and GaitBase [33], which achieve 46.3% and 44.0%, respectively. In addition, the Rank-5, Rank-10, and Rank-20 accuracies of GaitRGA also show a similar trend, with values of 82.14%, 86.31%, and 89.35%, respectively, further highlighting its superior performance across all evaluation metrics. These results confirm that our model is highly effective in capturing gait features in complex real-world field scenes, and its ability to adapt to real-world gait recognition tasks is significantly better than existing methods. GaitRGA’s strong performance across all metrics indicates its robustness and its ability to handle challenges like viewpoint variations, occlusion, and diverse walking environments more effectively than current state-of-the-art models.

#### 4.3.2. Evaluate on Gait3D Dataset

The results on the Gait3D [32] clearly highlight the outstanding performance of the GaitRGA model in practical applications. As shown in Table 5, it achieves significant improvements compared to the latest existing gait recognition methods. These results highlight the continued research potential of silhouette-based gait recognition approaches in advancing the field of gait recognition. GaitRGA has achieved significant improvement in various key indicators. Specifically, GaitRGA achieved a Rank-1 accuracy of 61.23%, a Rank-5 accuracy of 80.51%, a mean Average Precision (mAP) of 53.64%, and a minimum Inverse Negative Penalty (mINP) of 35.81%. Compared with other contour-based methods such as GaitSet [20], GaitPart [16], DAGait [16], our method far outperforms them. GaitRGA is also significantly superior to skeletonization methods such as GaitGraph2 [39], GPGait [41], and SkeletonGait [25].

#### 4.3.3. Evaluate on SUSTech1k Dataset

Results from the SUSTech1k [35] demonstrate the comprehensive performance of GaitRGA in multi-scene gait recognition. As shown in Table 6, GaitRGA achieves an overall accuracy of 59.72% across multiple scenes, highlighting its effectiveness in handling complex environments. The table presents a detailed comparison of the recognition accuracy across different scenes, where GaitRGA consistently outperforms existing methods in both challenging and controlled environments.In particular, the model shows a strong performance in Scene Carrying, achieving an accuracy of 59.85%. Additionally, in more complex scenes like Scene Umbrella and Scene Uniform, where factors such as lighting variations and occlusions are prevalent, GaitRGA still maintains an impressive recognition accuracy of 58.21% and 58.74%, respectively. These results underline the robustness of GaitRGA in real-world scenarios, where environmental variations play a significant role.

### 4.4. More Experiments

**Figure 8:**
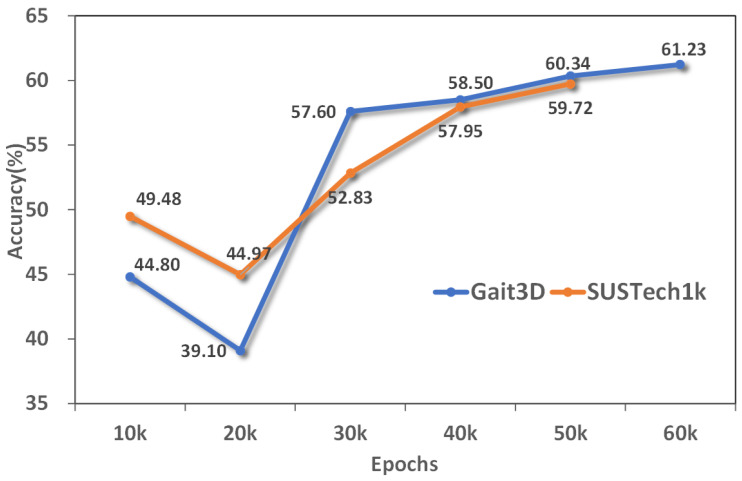
Rank-1 accuracy of GaitRGA at different rounds on Gait3D and SUSTech1k datasets.

**Figure 9:**
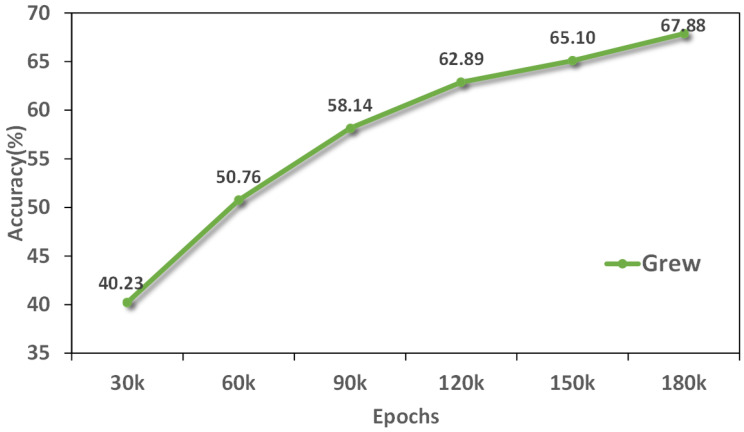
Rank-1 accuracy of GaitRGA at different rounds on Grew datasets.

We explored the Rank-1 accuracy changes of the three data sets under different training rounds. With the increase of training rounds, the accuracy of the two data sets is gradually improved and tends to be stable, indicating that the model is converging gradually. As shown in Figure 8, The accuracy of Gait3D data set increased steadily, while SUSTech1k showed large fluctuations and eventually stabilized at a higher accuracy. As shown in Figure 9, The accuracy of the Grew data set continued to improve and eventually converged. In general, with the increase of training rounds, the speed of accuracy improvement gradually slows down, indicating that the learning effect of the model tends to be saturated and close to its upper limit of performance.

Compared with GaitBase’s 19.29030 M, GaitRGA’s 20.01906 M is 0.72876 M longer, but the Rank-1 accuracy on Gait3D and Grew data sets is 6.53% and 7.78% higher than GaitBase’s, respectively. GaitRGA has achieved a significant improvement in performance compared to GaitBase, although the number of parameters is only increased by about 0.7 M. This shows that the GaitRGA model achieves a good balance between the parameter efficiency and the accuracy of gait recognition. By introducing innovative designs such as the global attention mechanism, GaitRGA can enhance the ability to extract and distinguish gait features effectively while maintaining relatively few model parameters, especially in the case of complex gait changes and different data sets.

This improvement is not only due to the simple increase in model complexity, but also due to the feature learning capability optimized by GaitRGA, which enables the model to better capture key dynamic information in gait, especially in the face of changing gait data. This result proves that GaitRGA not only performs well in the utilization of computing resources, but also shows a good prospect in the improvement of accuracy, showing its potential as a new generation model in the field of gait recognition.

## 5. Ablation Studies

### 5.1. Evaluate on RGA-S

After adaptively extracting the edge information of the gait silhouette through the RGA-S module, a performance improvement of 3.14% is achieved on Rank-1, 2.65% on Rank-5, and 2.38% on Rank-10. This demonstrates that RGA-S has strong robustness and discriminability in capturing the spatial relationships between prominent features.

### 5.2. Evaluate on RGA-C

The RGA-C module demonstrated stronger feature expression ability, and achieved a significant improvement of 4.63% on Rank-1, and 1.67% on rank-5, 1.37% on rank-10, and 1.28% on rank-20 after the RGA-S module. The performance advantage of this module is better than RGA-S in all evaluation indexes, and this phenomenon verifies the importance of multi-scale feature fusion in gait recognition from the side, demonstrating its ability to effectively capture the spatio-temporal dependencies in gait sequences, and provide richer feature representations for the difficult sample recognition.

### 5.3. Evaluate on RGA-S and RGA-C

As shown in Table 7. Notably, the combination of RGA-S and RGA-C achieves a Rank-1 accuracy of 67.88%, representing a 7.78% improvement over GaitBase. This improvement exceeds the individual contributions of the two modules when applied separately. This clearly demonstrates that the edge-aware mechanism and multi-channel feature fusion are complementary in the feature space, and the combination of the two can effectively integrate the overall motion patterns and local details of the features in the gait sequence in order to comprehensively capture the features during dynamic changes.

## 6. Conclusions

Conclusion This paper presents a Silhouette Relation-Aware Global Attention (Silhouette RGA) module optimized for gait silhouette images, which effectively addresses the challenges of noise interference and insufficient feature representation in gait recognition. Unlike traditional attention mechanisms, our approach not only learns attention through local convolutional operations but also comprehensively considers the global structural information between features. By simultaneously exploring relationships in both spatial and channel dimensions, our RGA module can more accurately capture discriminative features in gait silhouettes. Specifically, the Spatial Attention (RGA-S) module highlights the structural information of gait silhouettes through an edge enhancement mechanism, effectively extracting body part features in walking postures. Meanwhile, the Channel Attention (RGA-C) module focuses on the relationships between different feature channels, enhancing the ability to express dynamic gait patterns. By stacking these two attention mechanisms, the model forms an end-to-end network structure that effectively suppresses background interference and enhances perception of key body parts. Extensive experiments demonstrate that our proposed Relation-Aware Global Attention module achieves significant performance improvements compared to baseline models, proving the importance of global structural information for extracting discriminative features in gait. This approach does not require additional human semantic information (such as pose or segmentation masks) and can effectively learn more robust feature representations relying solely on gait silhouette images, providing a simple yet effective solution for gait recognition in practical applications.Future work will further explore the modeling of gait sequence relationships across time scales and how to more effectively incorporate richer appearance invariant features to further improve the robustness and adaptability of the model in complex environments. We will continue to optimize the model structure in subsequent studies to address this challenge.

## Figures and Tables

**Table 1:** ResNet-like backbone structure, where *k*, *c*, and *b* represent the kernel size, number of channels, and the number of layers, respectively.

Layer	Structure [k×k,c]×b
Conv0	[3×3,64]×1,stride=1
Layer1	$\left[\begin{array}{c} 3\times 3,64\\ 3\times 3,64 \end{array}\right]\times 1,\;\mathrm{stride}=1$
Layer2	$\left[\begin{array}{c} 3\times 3,64\\ 3\times 3,128 \end{array}\right]\times 1,\;\mathrm{stride}=2$
Layer3	$\left[\begin{array}{c} 3\times 3,128\\ 3\times 3,256 \end{array}\right]\times 1,\;\mathrm{stride}=2$
Layer4	$\left[\begin{array}{c} 3\times 3,256\\ 3\times 3,512 \end{array}\right]\times 1,\;\mathrm{stride}=1$

**Table 2:** Number of IDs and Seqs in the Grew, Gait3D, and SUSTech1k.

	Train		Test	
**Datasets**	**#ID**	**#Seq**	**#ID**	**#Seq**
Grew	20,000	102,887	6000	24,000
Gait3D	3000	18,940	1000	6369
SUSTech1k	200	5988	850	19,228

**Table 3:** Hyperparameter settings for experiments with three different datasets.

Dataset	BatchSize	Steps	Mutistep Scheduler
Grew	(32, 4)	180,000	(80 k, 120 k, 150 k)
Gait3D	(32, 4)	60,000	(20 k, 40 k, 50 k)
SUSTech1k	(8, 8)	50,000	(20 k, 40 k)

**Table 4:** Comparison of GaitRGA with existing SOTA methods on the Grew dataset.

Input	Method	Venue	Rank-1	Rank-5	Rank-10	Rank-20
	GaitSet [20]	AAAI’19	46.3	63.6	70.3	76.9
	GaitPart [16]	CVPR’20	44	60.7	67.4	73.5
	GaitGL [15]	ICCV’21	51.4	67.5	72.8	77.3
Silhouette-based	GaitBase [33]	CVPR’23	60.1	75.5	80.4	84.1
	GaitSSB [38]	CVPR’23	61.7	-	-	-
	QAGait [19]	AAAI’24	59.1	74	79.2	83.2
	**GaitRGA**	**Ours**	**67.88**	**82.14**	**86.31**	**89.35**
	GaitGraph2 [39]	CVPRW’22	33.5	49.4	56.2	61.9
Skeleton-based	Gait-TR [40]	ES’23	54.5	-	-	-
	GPGait [41]	ICCV’23	53.6	-	-	-
Multi-model	GairRef [42]	IJCB’23	53	67.9	73	77.5
	GaitSTR [43]	CVPR’24	64	78.5	83.2	86.3

**Table 5:** GaitRGA’s performance was assessed on the Gait3D dataset using metrics such as Rank-1, Rank-5, mAP, and mINP.

Input	Method	Venue	Rank-1	Rank-5	mAP	mINP
	GaitSet [20]	AAAI’19	36.7	58.3	30	17.3
	GaitPart [16]	CVPR’20	28.2	47.6	21.6	12.4
	GaitGL [15]	ICCV’21	29.7	48.5	22.3	13.6
Silhouette-based	GaitBase [33]	CVPR’23	54.7	-	-	-
	DANet [44]	CVPR’23	48	69.7	-	-
	**GaitRGA**	**Ours**	**61.23**	**80.51**	53.64	35.81
	GaitGraph2 [39]	CVPRW’22	11.1	24	-	-
Skeleton-based	Gait-TR [40]	ES’23	6.6	-	-	-
	GPGait [39]	ICCV’23	53.6	-	-	-
	SkeletonGait [25]	CVPR’24	38.1	56.7	28.9	16.1
	GairRef [42]	IJCB’23	53	67.9	**73**	**77.5**
Multi-model	SMPLGait [32]	CVPR’24	38.1	56.7	28.9	16.1
	HybridGait [18]	AAAI’24	53.3	72	43.3	26.7

**Table 6:** Rank-1 accuracy comparison on SUSTech1K with other leading models.

Method	Venue	Normal	Bag	Clothing	Carrying	Umbrella	Uniform	Occusion	Night	Overall
GaitPart [16]	CVPR’19	62.2	**62.8**	**33.1**	59.5	57.2	54.8	57.2	21.7	59.5
Gait-TR [40]	ES’23	22.2	18.2	6.8	18.6	13.4	19.2	27.3	16.4	18.6
GaitGraph2 [39]	CVPRW’22	33.3	31.5	21	30.4	22.7	34.3	44.9	23.5	30.8
**GaitRGA**	**Ours**	**65.70**	62.43	30.01	**59.85**	**58.21**	**58.74**	**60.82**	**22.17**	**59.72**

**Table 7:** The ablation experiment was conducted on the dataset with GaitBase as the Baseline, and Rank-K (K = 1, 5, 10, 20) as the evaluation index.

Method	RGA-S	RGA-C	Rank-1	Rank-5	Rank-10	Rank-20
			60.1	75.5	80.4	84.1
Baseline	✓		63.24	78.15	82.78	86.1
		✓	64.7	79.82	84.11	87.47
	✓	✓	**67.88**	**82.14**	**86.31**	**89.35**

## Data Availability

Gait3D, Grew and SUSTech1k datasets all need to be applied to third parties, and we cannot disclose detailed data.
